# Aseptic Viral Meningitis Secondary to Herpes Simplex Virus 2 Genital Infection

**DOI:** 10.7759/cureus.14535

**Published:** 2021-04-17

**Authors:** Camille Marshall, Zachary T Clark, Michael R Minckler

**Affiliations:** 1 Biology, Whitman College, Walla Walla, USA; 2 Molecular Biology, Whitman College, Walla Walla, USA; 3 Emergency Medicine, Providence St. Mary Medical Center, Walla Walla, USA

**Keywords:** viral meningitis, aseptic meningitis, herpes simplex virus, immunocompromised

## Abstract

Aseptic viral meningitis is the most common cause of meningitis in the United States. Most cases of herpes simplex virus meningitis are caused by herpes simplex virus type 2 (HSV-2), with HSV-1 primarily causing viral encephalitis. In this report, we present a case of aseptic meningitis generated by the HSV-2 in an immunosuppressed 35-year-old female with a recent diagnosis of genital herpes that was left untreated due to reported side effects of medication.

## Introduction

As the prevalence of compromising immunological conditions increases, the incidence of viral meningitis (VM) including herpes simplex virus type 2 (HSV-2) will also likely increase. These compromising conditions may include an aging population and diabetes. In 2003, the Centers for Disease Control and Prevention estimated that VM results in between 26,000 and 42,000 hospitalizations each year [1]. The incidence of VM in the United States has been estimated to be 0.26-17 cases per 100,000 people [2]. A prospective cohort study published in 2018 by Fiona McGill et al. showed that in United Kingdom adults, 55% of VM cases were caused by enteroviruses, followed by HSV as the second most common cause at 44% [3]. Such cases must be recognized and treated promptly in order to avoid increased complications and possible neurologic sequelae [4-6]. Neurologic sequelae of VM, such as chronic headaches, short-term memory loss, paresthesia or sensory deficits, and discoordination, can be severe in the short and long term [3,4,7]. While antivirals are currently recommended for treatment of viral encephalitis, VM treatment with acyclovir and antivirals remains controversial and inconsistent. Supportive care remains the most common provision of treatment, as many patients may recover on their own [4]. However, Noska et al. suggest that neurologic sequelae could be decreased in immunocompromised patients with meningitis, from either HSV serotype, if they were treated with antivirals [3,4,8].

## Case presentation

A 35-year-old female presented to the emergency department (ED) complaining of neck stiffness for one week, which had worsened over the previous 24 hours, and a gradual-onset headache. The pain was non-radiating, worse with both active and passive flexion, and rated at 10 out of 10. The patient had tried taking ibuprofen, which provided no relief. The patient also reported body aches, chills, photophobia, and had a fever of 100.8 F.

The patient has an underlying history of HSV-2. Two weeks ago, she was newly diagnosed with a genital herpes infection, which was likely contracted from her new sexual partner three months ago. She was prescribed oral acyclovir; however, she declined treatment due to reported facial swelling as a side effect due to acyclovir while taking it for shingles. Other medical history included acute myelogenous leukemia in remission since 1998, shingles, poorly controlled type 1 diabetes mellitus, and protein S deficiency. Her current medication included insulin aspart, alprazolam, ondansetron, and topical lidocaine. Social history was significant for a new sexual partner within the previous three months. She denied any tobacco, alcohol, or drug use.

Initial vital signs were a temperature of 100.8 F, heart rate of 115, respiratory rate of 18, blood pressure of 152/96 mmHg, and SpO2 of 97%. Significant physical examination findings included nuchal rigidity, swollen lymph nodes in groin area, and vesicular vaginal lesions.

Lab workup that was within normal limits included a complete blood count with differential, complete metabolic panel, lactate, negative urine human chorionic gonadotropin test, and urinalysis. Her serum glucose was 215 mg/dL, which was consistent with her history of poorly controlled type 1 diabetes mellitus. A lumbar puncture was performed, which revealed a cerebral spinal fluid (CSF) glucose of 99 mg/dL (reference range is >41 mg/dL), protein level of 80 mg/dL (reference range is 15-45 mg/dL), a negative gram stain, 4+ white blood cells, and absolute white blood cell count was 8.2 k/uL without left shift. CSF was sent for culture and nucleic-acid amplification testing (NAAT) analysis for HSV and varicella zoster virus. Blood samples were also sent for bacterial culture and came back negative. A head computed tomography (CT) was unremarkable. The patient’s Glasgow Coma Scale score was 15, she clinically appeared to have meningitis, and there was not clinical evidence of encephalitis.

Due to the patient history, clinical presentation, CSF findings, and an immunocompromised state, the patient was admitted to the hospital and initially started on intravenous acyclovir, broad-spectrum antibiotics, and deep vein thrombosis prophylaxis. The patient had reported mild facial swelling from previous treatment with acyclovir; the risks and benefits were weighed and intravenous acyclovir was selected as the best option. The patient’s vital signs stabilized within 24 hours of beginning treatment and her headache severity level improved to 1 out of 10. NAAT analysis of CSF returned positive for HSV-2. The patient steadily improved over her hospital stay, there were no sequelae, and was eventually discharged home after five days with home healthcare follow-up treatment for intravenous acyclovir treatment.

## Discussion

When considering the ED evaluation of central nervous system infections, two of the most concerning differential diagnoses include bacterial meningitis (BM) and HSV-1 encephalitis. BM and HSV-1 may progress rapidly, leading to poor neurologic outcomes [7,9]. While VM is also considered, most cases do not invoke concern for permanent sequelae as the disease course from the causative organisms is generally self-limited [3].

In a patient with poorly controlled diabetes mellitus type 1, an immunocompromised state promoted the meningeal seeding of her genital HSV-2 infection. Literary sources have identified that untreated HSV-2 meningeal infections may lead to significant morbidity and mortality [3,4,10]. The risk of suffering permanent disability from this disease is even greater in immunocompromised patients [10]. Immunocompromised patients are found to have more neurologic sequelae following HSV meningitis than immunocompetent patients [4]. Therefore, it would be prudent for ED physicians to increase their index of suspicion and consider prompt evaluation and therapy for HSV-2 meningitis in any immunocompromised adult with documented HSV-2 infection.

Furthermore, in the outpatient setting, a genital HSV-2 infection is often self-limited in otherwise healthy individuals. Therefore, treatment is often considered elective. Additionally, the role of antivirals to treat meningitis is still unclear [4]. However, this case demonstrates the need to initiate anti-viral treatment for patients who have active HSV-2 genital infections and a low-functioning immune system.

## Conclusions

With population health trends and current evidence, VM may become an increasing concern in years to come. More investigation will need to be done regarding which circumstances lend to early aggressive therapy versus a watch-and-wait approach when it comes to genital HSV-2 outbreaks. Future research will need to elucidate if there are significant differences in risk between first or subsequent outbreak of HSV-2 as well as comorbidities and their time courses that are causing the immunocompromised state. Currently, we advocate for a lower threshold to treat in the outpatient setting and increased index of suspicion in the ED.
